# Analysis of disease burden in patients with hereditary angioedema from Japan by patient‐reported outcomes

**DOI:** 10.1111/1346-8138.17421

**Published:** 2024-09-11

**Authors:** Michihiro Hide, Miwa Kishimoto, Ippei Kotera, Akinori Oh, Yoichi Inoue, Beverley Anne Yamamoto, Shinichi Noto

**Affiliations:** ^1^ Department of Dermatology, Hiroshima City Hiroshima Citizens Hospital Hiroshima Japan; ^2^ Department of Dermatology Hiroshima University Hospital Hiroshima Japan; ^3^ Japan Medical Office Takeda Pharmaceutical Company Limited Tokyo Japan; ^4^ Hereditary Angioedema Japan (HAEJ) Registered Non‐Profit Organization Hyogo Japan; ^5^ Graduate School of Human Sciences Osaka University Osaka Japan; ^6^ Department of Rehabilitation, School of Occupational Therapy Niigata University of Health and Welfare Niigata Japan

**Keywords:** hereditary angioedema, patient‐reported outcome measures, quality of life, surveys and questionnaires

## Abstract

Hereditary angioedema (HAE) symptoms can vary greatly. Disease burden evaluation is essential for providing adequate treatments for patients. Patient‐reported outcome measures (PROMs), including the 12‐Item Short Form Health Survey (SF‐12), the Angioedema Quality of Life (AE‐QoL), the Hospital Anxiety and Depression Scale (HADS), and the Work Productivity and Activity Impairment: Specific Health Problem (WPAI:SHP) questionnaires, were collected in 2021, before modern medications for long‐term prophylaxis (LTP) of HAE were licensed in Japan. Patients also reported their HAE attack frequency as “annual” (several attacks annually), “monthly” (several attacks monthly) or “weekly” (several attacks weekly). Multiple linear regression analyses were conducted on the relationship between independent parameters (sex, age, attack frequency, HAE type, and HADS scores) and dependent parameters (AE‐QoL and SF‐12 scores). Fifty‐four patients reported PROMs. All PROMs showed substantial health‐related quality of life (HRQoL) impairment. Overall, the higher the attack frequencies, the greater the reported impairment in the PROMs tended to be. In multiple linear regression analyses, higher AE‐QoL Fatigue/Mood and Fears/Shame domain scores (greater impairment) were associated with higher HADS anxiety subscale scores; higher AE‐QoL total scores (greater HRQoL impairment) and lower SF‐12 Physical and Mental Health Composite scores (greater general health impairment) were associated with higher HADS depression subscale scores. Patients with monthly or weekly HAE attacks reported numerically low absenteeism and numerically high presenteeism and work productivity loss as measured by the WPAI:SHP questionnaire. In this study, conducted before modern LTP options were available in Japan, patients with HAE reported notable impairment in HRQoL and work productivity. Weekly or monthly HAE attack frequencies were associated with a high disease burden. Furthermore, a substantial number of patients reported notable fatigue/mood impairment as measured by the AE‐QoL and depression as measured by the HADS regardless of attack frequency. These results provide a basis for future studies evaluating the effect of LTP on the clinical manifestations and HRQoL in patients with HAE.

## 1 INTRODUCTION

Hereditary angioedema (HAE) is a rare genetic disorder characterized by the paroxysmal appearance of swelling in various parts of the body due to increased vascular permeability.^1^, ^2^ HAE is primarily a deficiency in functional C1 esterase inhibitor (C1‐INH); HAE Type I is characterized by low levels of both C1‐INH protein and its functional activity, while HAE Type II presents with normal levels and a dysfunction of C1‐INH protein. HAE with normal C1‐INH (nC1‐INH‐HAE), historically referred to as “HAE Type III”, is a rare subtype of HAE, which shows similar manifestations of clinical symptoms with no mutation or defect in the C1‐INH gene (*SERPING1*).^3^, ^4^, ^5^ To date, mutations associated with nC1‐INH‐HAE have been identified in the following eight genes: factor XII (*F12*), angiopoietin‐1 (*ANGPT1*), plasminogen (*PLG*), kininogen 1 (*KNG1*), myoferlin (*MYOF*), heparan sulfate‐glucosamine 3‐*O*‐sulfotransferase 6 (*HS3ST6*), carboxypeptidase N (*CPN1*), and disabled homolog 2‐interacting protein (*DAB2IP*).^5^, ^6^, ^7^ The global prevalence of HAE Type I/II is estimated to be one in 50 000 individuals,^5^ and the prevalence of nC1‐INH‐HAE is estimated to be much lower than that of HAE Type I or II.^8^

Not only the severity and frequency, but also the location of HAE attacks may vary among patients.^9^ Common HAE manifestations include subcutaneous edema, mucosal edema, laryngeal edema, and intra‐abdominal edema, which contributes to severe abdominal pain, distension, cramping, and nausea.^10^ If the patient has not been diagnosed with HAE, severe episodes of gastrointestinal edema can result in an unnecessary laparotomy.^11^ Laryngeal edema is the most serious and life‐threatening symptom of HAE because of the risk of asphyxiation.^12^ The various manifestations of the disease and their unpredictability result in physical, mental, and socioeconomic burdens on patients.^8^, ^13^

The standard treatment for HAE includes on‐demand therapy (ODT), short‐term prophylaxis (STP), and long‐term prophylaxis (LTP).^5^, ^14^ Historically, the main goal of HAE treatment was to alleviate the attack symptoms through on‐demand drug administration. However, the current international World Allergy Organization (WAO)/European Academy of Allergy and Clinical Immunology (EAACI) guidelines for the management of HAE recommend that the goals of HAE treatment are to achieve total control of the disease and to normalize patients’ lives.^5^ Currently, these goals can be achieved only through LTP, by the regular administration of drugs to prevent attacks and, thereby, reduce disease burden.

In Japan, two kallikrein inhibitors, berotralstat and lanadelumab, were approved as LTP options in January 2021^15^ and March 2022,^16^ respectively. Subcutaneous C1‐INH was approved as an LTP option in Japan in September 2022.^17^ Considering the high treatment costs of these drugs and the variety of HAE symptoms, patient suitability for the new LTP options should be carefully assessed based not only on medical symptoms, but also on an accurate understanding of patient health‐related quality of life (HRQoL). This process should include physician, patient, and caregiver perspectives on issues such as HRQoL, treatment preference, mental burden, and work productivity.

Patient‐reported outcome measures (PROMs) are based on the use of self‐reported questionnaires without the need for assistance by health‐care professionals.^18^ PROMs are used to assess patients’ health status, HRQoL, and functional status associated with health care or treatment.^19^ Comprehensive generic PROMs, such as the 36‐Item Short‐Form Health Survey (SF‐36)^20^ and the 12‐Item Short‐Form Health Survey, version 2.0 (SF‐12),^21^ are suitable for assessing HRQoL in patients with any illness, and for comparing patient HRQoL with that of healthy subjects. However, generic PROMs may not adequately assess disease‐specific HRQoL impairment. Two disease‐specific PROMs have been developed to evaluate HRQoL in patients with recurrent angioedema: the Angioedema Quality of Life (AE‐QoL) questionnaire,^22^, ^23^ which was subsequently validated in adult patients with HAE,^24^ and the Hereditary Angioedema Quality of Life (HAE‐QoL) questionnaire.^25^, ^26^ Additionally, the Angioedema Activity Score (AAS) can be used to evaluate angioedema attacks using angioedema attack duration, severity, and impact on daily activities as reported by the patient.^23^, ^27^ Among those measures, the AE‐QoL and the AAS have been validated in Japanese.^23^

It is known that patients with HAE are prone to anxiety and depression.^28^ Concern about attacks may affect patients’ education and career, even during periods without attacks.^29^ Therefore, the assessment of long‐term HRQoL in patients with HAE, covering mental symptoms, productivity, and activity impairment, is essential.^30^ The Hospital Anxiety and Depression Scale (HADS)^31^, ^32^ is a comprehensive tool assessing anxiety and depression over the past week without asking the patients about direct symptoms of the disease. The Work Productivity and Activity Impairment V2.0: Specific Health Problem (WPAI:SHP) questionnaire^33^, ^34^, ^35^ measures the effect of health problems on patients' ability to work and participate in regular activities.

Associations between higher HAE attack frequency and impairments in comprehensive generic PROMs, disease‐specific PROMs, and WPAI scores have been reported.^36^, ^37^ However, the reported effect of LTP on the HRQoL, especially the psychological aspects, in patients with HAE is not consistent between different studies. A survey of patients from the United States, most of whom (68.5%) had received or were receiving LTP, reported that patients having no attack during the past 6 months had the lowest anxiety and depression scores as measured by the HADS; patients having 13 or more attacks had the highest scores.^36^ However, a multinational survey showed moderate to severe anxiety and depression in 38.0% and 17.4% of patients regardless of attack frequency.^37^ Moreover, a long‐term study of a new LTP medication showed a significant HRQoL improvement in patients, two‐thirds (63%) of whom had been on prophylactic treatment prior to that study.^38^ Of note, mean baseline values of participants in that study were already better than US population norms for the EQ‐5D (Health State Value and Visual Analogue Scale), and within the scoring range interpreted as normal for HADS scores.^38^

These points highlight the importance of evaluating the impact of LTP on HRQoL in patients with HAE by detailed and comprehensive assessment including mental status (e.g., anxiety and depression) as well as physical symptoms, both during and between attacks.

Previous studies focusing on the HRQoL of patients with HAE were mainly conducted in patients of European descent living in North America or Western Europe who have access to LTP treatments, approximately 40%–60% of these patients receiving C1‐INH and approximately 30% receiving attenuated androgens.^37^, ^38^ There is less information on HRQoL in patients with HAE from East Asian populations who have limited access to the modern effective treatments for LTP recommended by global guidelines.^5^, ^39^, ^40^, ^41^

At the time of this survey, both plasma‐derived C1‐INH and icatibant were approved in Japan for STP and/or treating acute attacks. Danazol, an attenuated androgen, was licensed for female endometriosis but not for HAE and, therefore, was rarely taken for HAE as an off‐label use.^40^ Prescribing tranexamic acid for HAE has been relatively easy in Japan because of its license for urticaria, which may accompany other forms of angioedema. However, the efficacy of tranexamic acid as ODT or LTP in HAE is very limited.^42^ Previous reports on HRQoL in patients with HAE from Japan included data collected before icatibant was licensed for ODT of HAE attacks in 2018 and patients could access effective treatment only at a hospital.^40^, ^41^

Our survey captured, for the first time, HRQoL in patients with HAE in Japan after self‐administered ODT became available, but effective LTP was still absent, with the expectation that having access to self‐administered ODT would bring patient control to an unpredictable disease and lower the disease burden regardless of medication use frequency. The aim of this study was to quantify HRQoL in patients with HAE by assessing generic and disease‐specific PROMs, mental status, and work productivity.

## 2 METHODS

Study participants were recruited from February 2021 to June 2021 through two HAE patient advocacy groups in Japan: Hereditary Angioedema Japan (HAEJ),^43^ a registered non‐profit patient organization, and the patient advocacy group Kumi‐mu.^44^ An e‐mail announcement of this survey was sent to the members of these advocacy groups. Patients in these groups could participate in the study by clicking on the independently provided questionnaire link, which led to the website containing the study information and eligibility criteria necessary for participation. After a review of the information and agreement to the informed consent statement, patients answered questions related to the inclusion and exclusion criteria for the screening process.

Patients were eligible for this study if they met the following criteria: ≥18 years of age, had a physician‐verified diagnosis of HAE (self‐reported), had received any treatment for HAE within the last 6 months, had not received any investigational agents in clinical trials of HAE at the time of the survey, and had a basic level of Japanese sufficient to understand and answer the questionnaire. Individuals under the age of 20 were allowed to participate in the survey if both the patient and their caregiver agreed to the informed consent statement. Participants who met the inclusion criteria during screening were then directed to answer the survey questions. Participants who did not meet the inclusion criteria during screening were not directed to the survey and any data collected up to that point were not included in the results.

The survey consisted of questions about demographic and clinical characteristics as well as medical and medication history and HRQoL, including age, sex, comorbidities, history of malignancy, psychiatric disorders, HAE type, age at HAE onset and HAE diagnosis, types of symptoms, sites and areas of HAE attacks, family history, and HAE treatment. Patients were asked to select the frequency of their HAE attacks from “annual” (defined as several HAE attacks annually), “monthly” (defined as several HAE attacks monthly), or “weekly” (defined as several HAE attacks weekly), and the HAE type from “HAE type 1 or 2” (HAE Type I/II), “HAE type 3” (nC1‐INH‐HAE), or “I don't know” (unsure of HAE type).

Detailed information about the PROMs used in this study is provided in Table S1. The SF‐12 is a validated instrument designed to measure functional health and well‐being.^21^ It evaluates general health during the past 4 weeks over 12 questions in eight domains (physical functioning, role‐physical, bodily pain, general health, vitality, social functioning, role‐emotional, and mental health) and two summary scores (Physical Health Composite and Mental Health Composite). SF‐12 domain and summary scores range from 0 to 100, where higher scores reflect better health and a score of 50 reflects the natural standard score of the general population.^21^ It has also been suggested that a SF‐12 Mental Health Composite score of ≤42 may be indicative of clinical depression.^45^ The use of SF‐12 in Japanese was licensed from Qualitest (Kyoto, Japan).

Disease‐specific HRQoL was evaluated using the AE‐QoL.^22^, ^23^ The AE‐QoL assesses HRQoL impairment in patients with recurrent angioedema during the past 4 weeks over 17 questions in four domains (Functioning, Fatigue/Mood, Fears/Shame, and Nutrition), and a total score.^22^, ^23^ AE‐QoL scores range from 0 to 100, where higher scores reflect a greater impairment in HRQoL; an AE‐QoL total score of ≥39 reflects moderate to large HRQoL impairment.^22^, ^46^

Anxiety and depression were assessed with the HADS.^31^, ^32^ The HADS is a generic measure assessing anxiety and depression during the past week using 14 questions (seven for anxiety and seven for depression). HADS subscale scores range from 0 to 21, where scores of 0–7 are suggestive of normal levels of anxiety/depression, scores of 8–10 are suggestive of mild anxiety/depression, and scores over 11 are suggestive of moderate to severe anxiety/depression (moderate score, 11–14; severe score, 15–21).^31^, ^47^ Furthermore, the HADS total score can be calculated by summing all HADS items; the HADS total score has a range of 0–42, with higher scores indicating greater psychological distress.^48^

Impairment in patients' ability to work and perform regular activities due to HAE was evaluated using the WPAI:SHP questionnaire.^33^, ^34^, ^35^ The WPAI:SHP assesses productivity loss during the past 7 days over six questions to calculate the percentage of impairment in absenteeism, presenteeism, overall work productivity loss, and activity impairment (in general daily non‐work activities).^34^, ^35^ WPAI:SHP scores range from 0 to 100, where higher scores reflect greater productivity loss.^34^, ^35^

Additionally, patients who reported an attack within 24 h before answering the study questionnaire were asked to report an AAS.^23^, ^27^ The AAS assesses angioedema activity in patients with recurrent angioedema in the past 24 h over five questions; AAS scores range from 0 to 15, with higher scores reflecting greater disease activity.^23^, ^27^

Descriptive analyses were performed to understand the quantitative characteristics of the patients who participated in the study. Categorical variables were analyzed with frequency tables and continuous variables were analyzed with summary statistics. PROMs were stratified by the frequency of HAE attacks: annual, monthly, and weekly. Multiple linear regression models included the following independent variables: sex, age, frequency of HAE attacks, type of HAE attacks, and the HADS items (anxiety and depression). The dependent variables were AE‐QoL (total score; Functioning, Fatigue/Mood, Fears/Shame, and Nutrition domain scores) and SF‐12 (Physical Health Composite and Mental Health Composite scores).

To ensure data anonymity, patients were guided to the questionnaire through individually distributed questionnaire links and no information that could reveal their identity was collected. The data were collected using SAP Qualtrics (version 082020 https://www.qualtrics.com). Aggregation and statistical analysis were performed in R (R: Version 4.0.5 2021, the R Foundation for Statistical Computing) and SAP Qualtrics.

This study was conducted in accordance with the 1964 Declaration of Helsinki and its later amendments. Ethics approval for this study was obtained from the Ethics Committee of the Non‐Profit Organization, MINS Research Ethics Committee (approval number: MINS‐REC‐200222).^49^ The study was registered in the UMIN Clinical Trials Registry (UMIN000042425) before enrollment of the first participant.

## 3 RESULTS

### 3.1 Patient demographics

**FIGURE 1:**
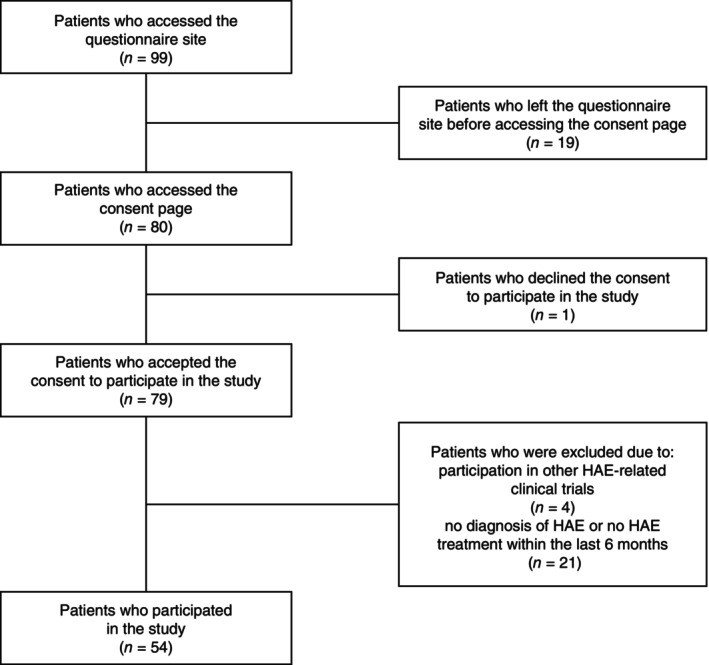
Flow chart of patient inclusion and exclusion.

Of the 99 patients who clicked on the questionnaire link, 54 met the inclusion criteria (see Methods) during screening and were included in this study. Details of the patient flow through the study are shown in Figure 1.

The key and detailed baseline characteristics of the patients are shown in Table 1 and Table S2, respectively. A breakdown of sex, age, gastrointestinal symptoms, and AE‐QoL by HAE subtype is provided in Table S3. Of 54 patients, 44 (81.5%) reported having HAE Type I/II, five patients (9.3%) reported having nC1‐INH‐HAE, and five patients (9.3%) reported being unsure of their HAE type despite having a physician‐confirmed diagnosis of HAE (Table 1). Forty‐seven (87.0%) of the 54 patients were female and seven (13.0%) were male. The mean ± SD age was 48.0 ± 12.1 years, and 72.2% of the patients were > 40 years of age at the time of the survey. The majority of patients (39/54; 72.2%) first experienced symptoms of HAE from 10 to 29 years of age; eight of 54 patients (14.8%) reported HAE symptom onset at <12 years of age, and 14 of 54 (25.9%) patients reported HAE symptom onset at 12–19 years of age. The mean ± SD age at the first symptom onset was 22.2 ± 12.5 years, and mean ± SD age at HAE diagnosis was 37.0 ± 12.9 years, indicating an average of 15 years of delay from onset to diagnosis (Table 1). Six of the seven male patients reported their attack frequency as annual. Four of the seven male patients reported having HAE Type I/II; one of these four patients had the age at onset in the range of 10–19 years, two had age at onset in the range of 20–29 years, and one patient had the age at onset in the range of 30–39 years. The other three male patients reported being unsure of their HAE type; one of these patients had an age at onset in the range of 10–19 years and the other two had an age at onset in the range of 20–29 years. All five of the patients with nC1‐INH‐HAE were female (Table S3). The symptoms of HAE attacks experienced by more than 50% of patients were subcutaneous and/or mucosal edema in 45 of 54 (83.3%), gastrointestinal symptoms in 40 of 54 (74.1%), and laryngeal edema (answered as “attacks in the throat”) in 30 of 54 (55.6%) patients (Table 1). The incidence of gastrointestinal symptoms by HAE subtypes is shown in Table S3. Regarding comorbidities, four of the five patients with urticaria had HAE Type I/II, and the fifth had nC1‐INH‐HAE (Table S2).

**TABLE 1:** Key demographic and clinical characteristics.

	All (*N* = 54)	Frequency of HAE attacks		
		Annual^a^ (*n* = 30)	Monthly^b^ (*n* = 15)	Weekly^c^ (*n* = 9)
Sex, *n* (%)				
Male	7 (13.0)	6 (20.0)	1 (6.7)	0 (0.0)
Female	47 (87.0)	24 (80.0)	14 (93.3)	9 (100.0)
HAE subtype, *n* (%)				
Type I or II	44 (81.5)	26 (86.7)	11 (73.3)	7 (77.8)
nC1‐INH‐HAE	5 (9.3)	0 (0.0)	4 (26.7)	1 (11.1)
Unknown status	5 (9.3)	4 (13.3)	0 (0.0)	1 (11.1)
Age, mean ± SD, years	48.0 ± 12.1	49.4 ± 13.6	46.0 ± 10.0	46.9 ± 10.8
Age at onset, mean ± SD, years	22.2 ± 12.5	25.0 ± 13.7	21.8 ± 9.3	13.3 ± 8.9
Age at diagnosis, mean ± SD, years	37.0 ± 12.9	38.8 ± 13.3	36.5 ± 10.6	31.7 ± 14.7
Symptoms of HAE attacks,^d^ *n* (%)				
Subcutaneous and/or mucosal edema	45 (83.3)	23 (76.7)	14 (93.3)	8 (88.9)
Gastrointestinal symptoms	40 (74.1)	17 (56.7)	14 (93.3)	9 (100.0)
Attacks on the throat	30 (55.6)	14 (46.7)	9 (60.0)	7 (77.8)

Abbreviations: C1‐INH, C1 esterase inhibitor; HAE, hereditary angioedema; nC1‐INH‐HAE, hereditary angioedema with normal C1 inhibitor; SD, standard deviation.

^a^ Annual: patients with several HAE attacks annually.

^b^ Monthly: patients with several HAE attacks monthly.

^c^ Weekly: patients with several HAE attacks weekly.

^d^ Multiple responses were allowed.

### 3.2 SF‐12

**FIGURE 2:**
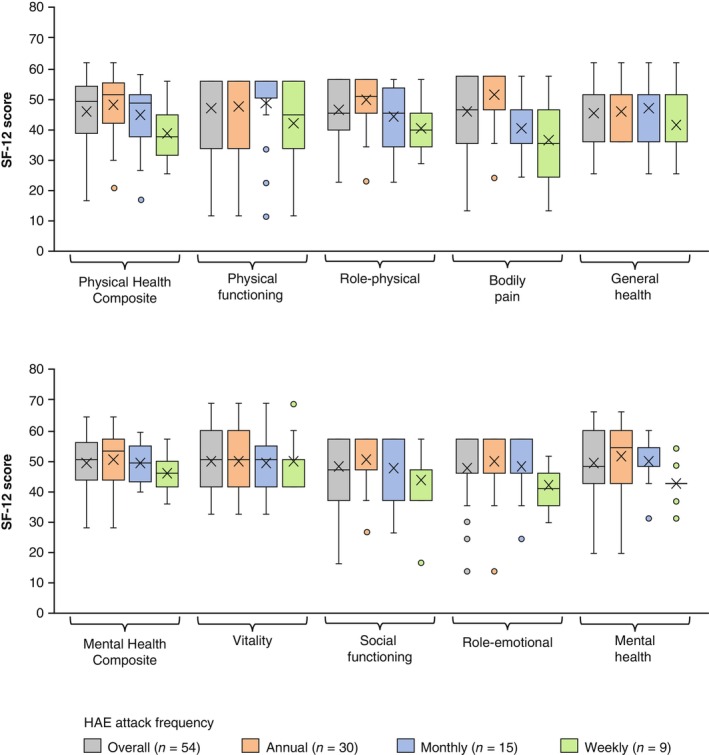
12‐Item Short Form Health Survey (SF‐12, version 2.0) scores by frequency of hereditary angioedema (HAE) attacks classified into annual, monthly, and weekly. Interquartile range was calculated using the inclusive median. Means are depicted by “x.”

Both of the SF‐12 summary scores (Physical Health Composite, mean of 45.8; Mental Health Composite, mean of 49.5), were below 50, which is the mean SF‐12 score in the general population, and showed a tendency to decrease with increasing attack frequency, suggesting greater HRQoL impairment in patients who reported more frequent HAE attacks. The numerical decrease in SF‐12 domain scores (greater HRQoL impairment) related to increasing attack frequency was particularly apparent in role‐physical, bodily pain, social functioning, role‐emotional, and mental health domains. The vitality domain scores were distributed around 50 regardless of attack frequency (Figure 2). Similar results were observed in an analysis that included only patients with HAE Type I/II and excluded the five patients with nC1‐INH‐HAE and five patients who were unsure of their HAE type (Figure S1).

### 3.3 AE‐QoL

**FIGURE 3:**
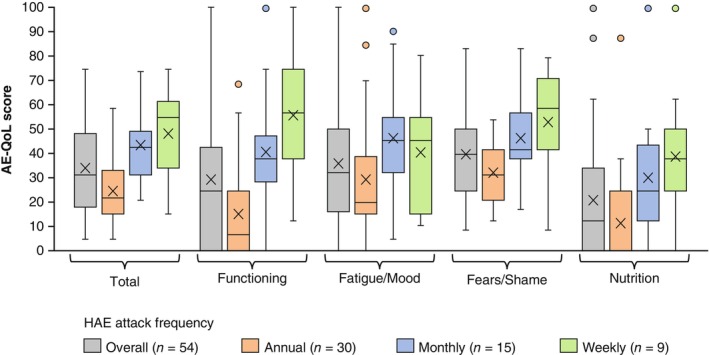
Angioedema Quality of Life (AE‐QoL) scores by frequency of hereditary angioedema (HAE) attacks classified into annual, monthly, and weekly. Interquartile range was calculated using the inclusive median. Means are depicted by “x.”

In the overall study population, AE‐QoL scores tended to increase, suggesting a greater HRQoL impairment, as the attack frequency increased. Patients who reported their attack frequency as monthly or weekly had AE‐QoL total scores higher than the threshold of ≥39, which is indicative of moderate to large HRQoL impairment.^46^ The AE‐QoL domains with the numerically highest scores were Fatigue/Mood and Fears/Shame (Figure 3). A similar tendency was observed in an analysis of patients with HAE Type I/II only, which excluded the 10 patients with nC1‐INH‐HAE or who were unsure of their HAE type (Figure S2).

### 3.4 AAS

**FIGURE 4:**
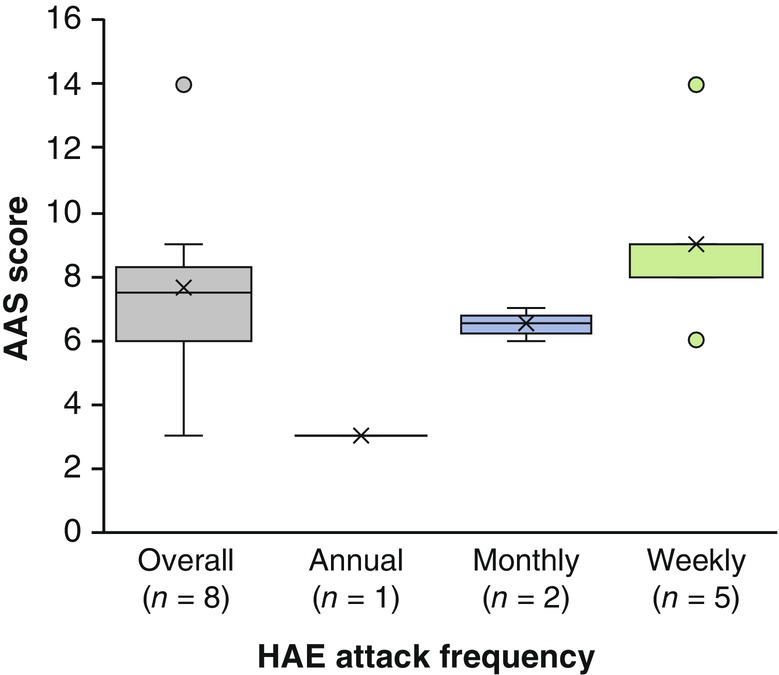
Angioedema Activity Score (AAS) scores by frequency of hereditary angioedema (HAE) attacks classified into annual, monthly, and weekly. Scores of AAS were collected from eight patients who reported an attack in the 24 h prior to answering the questionnaire.

Eight patients reported an attack within 24 h of the survey and were, therefore, asked to complete the AAS questionnaire part of the survey. The mean total AAS score of the respondents was 7.6. The AAS score tended to be higher, suggesting greater angioedema activity, in patients who reported their attack frequency as weekly versus those who reported their attack frequency as monthly or annual (Figure 4).

### 3.5 HADS

**FIGURE 5:**
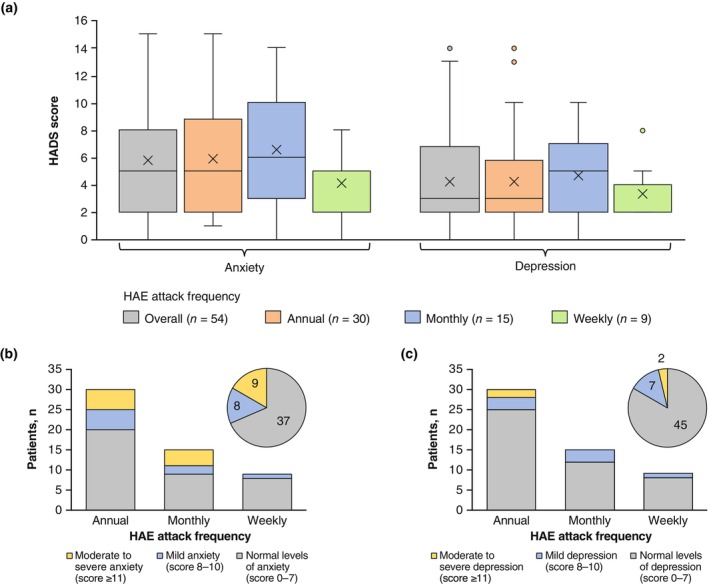
Hospital Anxiety and Depression Scale (HADS) scores. (a) HADS anxiety and depression subscale scores by frequency of hereditary angioedema (HAE) attacks classified into annual, monthly, and weekly. Interquartile range was calculated using the inclusive median. Means are depicted by “x.” Number of patients with (b) HADS anxiety subscale scores and (c) HADS depression subscale scores indicative of normal levels of anxiety or depression (0–7), mild anxiety or depression (0–8), and moderate to severe anxiety or depression (≥11). Circle graph inserts show overall distributions of the patients in HADS categories of anxiety and depression.

The HADS results are shown in Figure 5 and Figure S3. The mean anxiety and depression subscale scores in all patients were 5.8 and 4.2, respectively, and no apparent relationship was observed between HADS subscale scores and attack frequency. Seventeen of 54 (31.5%) patients had an HADS anxiety subscale score of ≥8 and nine of 54 (16.7%) patients had an HADS depression subscale score of ≥8, suggesting mild or moderate to severe anxiety or depression, respectively. Nine of 54 (16.7%) patients reported an HADS anxiety subscale score of ≥11, suggestive of moderate to severe anxiety, and two of 54 (3.7%) patients reported an HADS depression subscale score of ≥11, suggestive of moderate to severe depression.

### 3.6 WPAI:SHP

**FIGURE 6:**
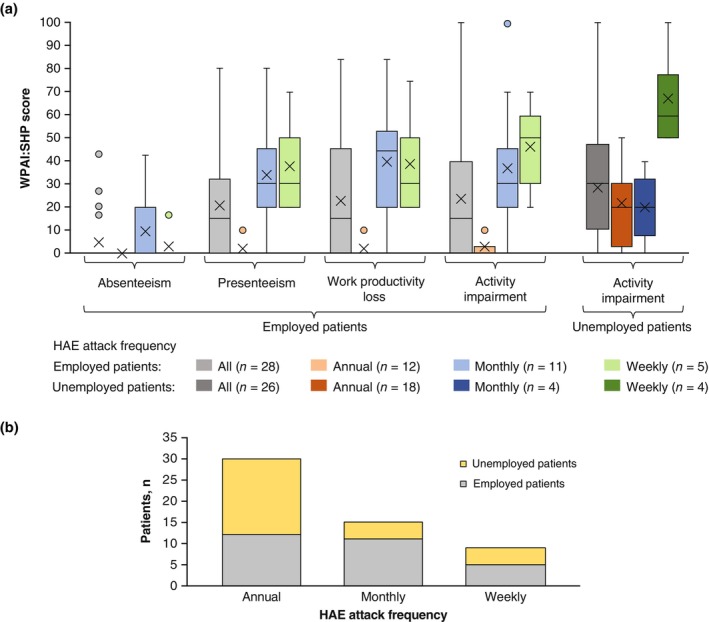
Work Productivity and Activity Impairment Specific Health Problem V2.0 (WPAI:SHP) scores by frequency of hereditary angioedema (HAE) attacks classified into annual, monthly, and weekly. (a) Percentage impairment in absenteeism, presenteeism, and work productivity loss was obtained from 28 patients with any employment. Percentage impairment in activity impairment was obtained from all patients in the survey and reported separately in employed and unemployed patients. Interquartile range was calculated using the inclusive median. Means are depicted by “x.” (b) Number of employed/unemployed patients by frequency of HAE attacks.

Twenty‐eight of 54 patients were employed. The mean percentage impairment in absenteeism, presenteeism, work productivity impairment, and activity loss of the employed patients were 4.5, 20.7, 23.2, and 23.9, respectively, indicating that employed patients had a tendency of low impairment in absenteeism and a tendency for high productivity loss in the other WPAI:SHP domains. Among the three attack frequency groups, a tendency was observed for low impairment in patients who reported their attack frequency as annual and high impairment in patients who reported their attack frequency as monthly or weekly (Figure 6a) in all WPAI:SHP domains. Of note, the activity impairment of the unemployed patients measured by the WPAI:SHP was similar or numerically higher than employed patients in all attack frequency groups. This result suggests that the burden of HAE may hinder patients in obtaining employment. Similar results were observed in an analysis of patients with HAE Type I/II only, which excluded eight employed and two unemployed patients with nC1‐INH‐HAE or who were unsure of their HAE type (Figure S4). The proportion of employed patients was numerically highest in patients who reported their attack frequency as monthly, followed by those who reported their attack frequency as weekly (Figure 6b).

### 3.7 Multiple linear regression analysis

The results of the multiple linear regression analysis are shown in Table 2. The AE‐QoL total score was significantly associated with attack frequency (both monthly and weekly) and the HADS depression subscale score. Among the AE‐QoL domains, Functioning and Fears/Shame domain scores were significantly associated with both monthly and weekly attack frequencies. The Nutrition domain score was associated with weekly attack frequency, and Fatigue/Mood domain score did not show any statistically significant association with attack frequency. Furthermore, higher AE‐QoL Fatigue/Mood and Fears/Shame domain scores (greater impairment in these domains) were significantly associated with higher HADS anxiety subscale scores (greater impairment in anxiety).

**TABLE 2:** Multiple linear regression analysis.

		AE‐QoL										SF‐12			
		Total		Functioning		Fatigue/Mood		Fears/Shame		Nutrition		Physical Health Composite		Mental Health Composite	
Predictor		(*N* = 54)		(*N =* 54)		(*N* = 54)		(*N =* 54)		*(N =* 54)		(*N =* 54)		(*N* = 54)	
		Coefficient	*p*	Coefficient	*p*	Coefficient	*p*	Coefficient	*p*	Coefficient	*p*	Coefficient	*p*	Coefficient	*p*
Demographics	Sex	−0.016	0.879	−0.028	0.815	−0.050	0.646	0.036	0.748	0.012	0.930	−0.075	0.482	−0.045	0.700
	Age	0.015	0.889	0.014	0.912	−0.010	0.927	−0.063	0.584	0.224	0.108	−0.254	0.023*	0.016	0.891
Frequency^a^ of HAE attacks	Monthly	0.357	0.004**	0.402	0.005**	0.179	0.155	0.298	0.024*	0.278	0.074	−0.338	0.007**	0.030	0.825
	Weekly	0.306	0.013*	0.496	<0.001***	−0.034	0.786	0.281	0.035*	0.327	0.040*	−0.387	0.003**	−0.070	0.608
Type of HAE attacks	Subcutaneous and/or mucosal edema	0.058	0.606	−0.008	0.949	0.141	0.241	0.050	0.683	−0.082	0.577	0.198	0.093	−0.113	0.377
	Gastrointestinal symptoms	0.106	0.354	0.106	0.430	0.143	0.245	−0.102	0.417	0.298	0.051	0.333	0.007**	−0.206	0.118
	Attacks on the throat	0.018	0.865	−0.018	0.886	−0.071	0.521	0.130	0.260	0.050	0.713	‐0.141	0.197	0.235	0.054
HADS	Anxiety	0.274	0.078	−0.037	0.838	0.416	0.014*	0.405	0.019*	−0.118	0.555	0.017	0.914	−0.241	0.172
	Depression	0.300	0.043*	0.327	0.059	0.278	0.076	0.137	0.391	0.180	0.344	−0.562	<0.001***	−0.360	0.034*
Model summary	Adjusted *R* ^2^	0.520		0.338		0.453		0.421		0.175		0.484		0.372	
	*F*‐statistic	7.380		4.002		5.882		5.286		2.250		6.528		4.488	
	*p*‐value	<0.001***		<0.001***		<0.001***		<0.001***		0.036*		<0.001***		<0.001***	

*Note*: Monthly and weekly attacks were defined as several HAE attacks monthly and weekly, respectively. Asterisks in the *p*‐value columns indicate level of statistical significance: *p < 0.05; **p < 0.01; ***p < 0.001.

Abbreviations: AE‐QoL, Angioedema Quality of Life Questionnaire; HADS, Hospital Anxiety and Depression Scale; HAE, hereditary angioedema; SF‐12, 12‐Item Short Form Health Survey (version 2.0).

^a^ Patients with annual attack frequency were used as a reference category.

The SF‐12 Physical Health Composite score was significantly associated with age, attack frequency (both monthly and weekly), gastrointestinal symptoms, and the HADS depression subscale score; the SF‐12 Mental Health Composite score was significantly associated only with the HADS depression subscale score. For both the SF‐12 Physical and Mental Health Composite, lower scores (greater impairment as measured by the SF‐12) were associated with higher HADS depression subscale scores (greater impairment in depression).

## 4 DISCUSSION

This study quantified the HRQoL of patients with HAE in Japan where the treatment of HAE is predominantly ODT, a unique situation not seen in other countries or regions. To extensively assess the HRQoL in Japan before the availability of modern LTP, multiple PROMs were used to evaluate general health status (SF‐12), disease‐specific HRQoL (AE‐QoL), angioedema activity (AAS), anxiety and depression (HADS), and productivity impairment (WPAI:SHP).

Both SF‐12 Physical and Mental Health Composite scores were below the general population mean score of 50. The mean AE‐QoL total scores in patients who reported their attack frequency as weekly and monthly were over 39, indicating moderate to large impairment of HRQoL.^46^ Moreover, domain scores of many SF‐12 and AE‐QoL domains tended to worsen with the increase of attack frequency. The multiple linear regression analysis also revealed significant associations between SF‐12 Physical Health Composite score, AE‐QoL total score, AE‐QoL Functioning domain score, AE‐QoL Fears/Shame domain score, and attack frequency categories. These results suggest that HRQoL in patients with HAE is substantially lower than the national standard, especially in patients who reported their attack frequency as monthly and/or weekly.

In the SF‐12, attack frequency‐dependent impairment tended to be more apparent in the role‐physical and bodily pain domains compared with physical functioning and general health domains. The AAS scores on the day of HAE attacks tended to be higher in patients who reported their attack frequency as weekly versus those who reported their attack frequency as monthly or annual (Figure 4). Furthermore, AAS scores were moderately associated with the SF‐12 role‐physical and bodily pain domains (data not shown). Thus, the role‐physical domain, which measures the limitations of daily activities imposed by physical burden, and the bodily pain domain may be more affected by the frequency and severity of HAE attacks compared with the SF‐12 physical functioning and general health domains. Additionally, the SF‐12 mental health domain score was also associated with attack frequencies. However, SF‐12 vitality domain scores were around 50 regardless of the attack frequency, possibly because the participants in this study were recruited through patient advocacy groups and might have maintained high levels of mental vitality despite burdens of HAE measured by other aspects.

Among the symptoms of HAE attacks, laryngeal and abdominal swellings were reported as the most excruciating manifestations for patients. Laryngeal swelling can be life‐threatening and may require intubation or, possibly, tracheotomy. Abdominal swellings are often accompanied by severe pain and may be misdiagnosed as peritonitis, potentially resulting in an unnecessary laparotomy.

A Chinese study found a significant association between laryngeal attacks and SF‐12 Mental Health Composite scores.^39^ However, our study showed no statistically significant relationship between AE‐QoL/SF‐12 scores and laryngeal attacks (reported as attacks on the throat). Likewise, no statistically significant relationship was found between the SF‐12 Mental Health Composite scores and gastrointestinal symptoms (Table 2). Additionally, a significant positive association was found between the SF‐12 Physical Health Composite scores and abdominal attacks, i.e., lower HRQoL impairment as measured by the SF‐12 Physical Health Composite score was associated with the presence of abdominal attacks.

Results of this study may, in part, reflect the different focus of the questions used in the studies. We asked about past attack experience, whereas the Chinese study focused on the current status of laryngeal edema. Moreover, the severity of symptoms was not captured in this study; thus, the severity of abdominal attacks reported by patients in our study might have been mild. The presence of a few patients without gastrointestinal symptoms showing very low scores in the SF‐12 Physical Health Composite score skewed the overall data of this group (Figure S5). The lack of an association between the AE‐QoL/SF‐12 scores and laryngeal attacks may imply a need for patient education to ensure that the patients fully understand that laryngeal HAE attacks should always be treated as life‐threatening, even if the patient experienced laryngeal attacks with mild symptoms in the past. The association of impairment in the AE‐QoL Nutrition domain with high HAE attack frequency may reflect severe attacks that hindered eating. Further research is needed to clarify these relationships.

Anxiety and depression are common clinical manifestations of patients with HAE.^28^ A survey conducted in the United States in 2017 reported an association between HAE attack frequency and anxiety/depression.^37^ However, a survey conducted in Australia, Canada, and six European countries (Austria, France, Germany, Spain, Switzerland, and the United Kingdom) in 2018 did not show such an association.^37^ In these studies, many patients were using intravenous injections of C1‐INH as LTP. In the current study, no apparent trend was observed between attack frequency and HADS scores. Patients who reported a monthly attack frequency showed numerically higher scores of the HADS anxiety and depression versus those who reported their attack frequency as weekly or annual (Figure 5). We further analyzed the relationships among AE‐QoL and SF‐12 domains and HADS subscales by a multiple linear regression analysis.

In the multiple linear regression analysis, higher AE‐QoL Fatigue/Mood and Fears/Shame domain scores (greater impairment as measured by the AE‐QoL) were associated with higher HADS anxiety subscale scores. It is not unexpected that patients with anxiety may experience fears and shame. However, the association of AE‐QoL Fatigue/Mood domain scores and anxiety in patients with HAE was neither expected nor reported previously. Considering that the AE‐QoL Fatigue/Mood domain score was not associated with the attack frequency, anxiety during the attack‐free periods may impact the patients’ fatigue and mood regardless of the presence of HAE attacks. Conversely, no specific AE‐QoL domain was associated with the HADS depression subscale score. However, a higher AE‐QoL total score (greater impairment as measured by the AE‐QoL) and lower SF‐12 Physical and Mental Health Summary scores (greater impairment as measured by the SF‐12) were associated with higher HADS depression scores. Moreover, nine of 54 patients had moderate to severe, and eight of 54 patients had mild anxiety as evaluated by the HADS anxiety subscale scores; additionally, two of 54 patients had moderate to severe, and seven of 54 patients had mild depression as evaluated by the HADS depression subscale scores (Figure 5). Taken together, these results suggest that regardless of the frequency of HAE attacks, patients with HAE should be monitored not only for anxiety associated with the feelings of fear and shame, but also for fatigue and depression.

Presenteeism and activity impairment as measured by the WPAI:SHP tended to worsen with an increasing frequency of HAE attacks. However, the mean percentage impairment for absenteeism and work productivity loss in patients who reported the frequency of their attacks as weekly was similar to or numerically lower than in patients who reported their attack frequency as monthly (Figure 6). A similar result was obtained in an analysis of patients with HAE Type I/II only, which excluded the 10 patients with nC1‐INH‐HAE or unsure of their HAE type (Figure S4). A similar observation was reported by Banerji, et al. in a study in the United States, where the mean score for absenteeism peaked at 7 to 12 attacks per 6 months and slightly declined for the group with more than 13 attacks per 6 months.^36^ This may be, at least partially, related to the fact that patients with a high frequency of HAE attacks may use ODT on a regular rather than an occasional basis, resulting in a frequency of medication use similar to that used for LTP. Notably, in the present study, a numerically high proportion of patients who reported their attack frequency as weekly used C1‐INH replacement therapy and icatibant (Table S2). It is also feasible that the patients who reported their attack frequency as weekly were not working full‐time, but instead were working in more flexible part‐time or remote jobs. In this study, the proportion of employed patients among those who reported their attack frequency as monthly and weekly (16 of 24, 66.7%) was numerically higher than that in patients who reported their attack frequency as annual (12 of 30, 40.0%) (Figure 6b). This issue should be investigated in future studies when LTP becomes widely available in Japan.

The WAO/EAACI international guidelines for the management of HAE recommend to evaluate patients for LTP at every visit, taking into consideration disease activity, impact on HRQoL, and disease control as well as patient preference.^5^ Reduction in the proportion of patients reporting anxiety and depression following LTP initiation has been reported in clinical studies.^50^ However, further data are needed on the effect of LTP treatments on anxiety and depression, since mental well‐being may be related not only to the current symptom severity under treatment with medications, but also to the history of attacks and the potential for breakthrough attacks. Therefore, it is preferable to compare the anxiety and depression of patients with LTP versus those without it over a sufficiently long span. In this study, we utilized the fact that modern LTP options recommended by the guidelines were unavailable at the time of data collection.

In this study, 33 of 54 (61.1%) patients reported that they had used tranexamic acid, and 16 (29.6%) patients reported that they had used plasma‐derived C1‐INH as a prophylactic agent in the last 6 months. However, tranexamic acid is not as effective as the three modern LTP options recommended in the guidelines.^5^, ^42^ Moreover, plasma‐derived C1‐INH was likely to be used for STP rather than LTP treatment, since it has been approved only for on‐demand and STP treatments in Japan. Androgen use was reported only in four of 54 (7.4%) patients. Thus, medications taken by the patients in this study as LTP should have minimal, if any, effect on the study results.

This study had several limitations. First, the number of patients was small, and their selection may have been biased. All 54 participants were selected from 99 patients recruited through two patient advocacy groups. The patients may have retained high activity due to their involvement in patient organizations, which may explain why SF‐12 vitality and social functioning domain scores did not show significant differences from those reported for the general population. Moreover, a large proportion of the patients (47 out of 54) were female. There were seven male participants in the study, of whom six reported their attack frequency as annual. Regarding the attack frequency, most of the participants (30/54 patients) reported their attack frequency as annual, whereas previous HAE studies in Japan had recorded higher overall attack frequencies.^40^ This may be because some of the patients who experienced monthly or more frequent HAE attacks were excluded from this study due to enrollment in clinical trials for HAE that were being conducted at the time of this study. The therapeutic drug use frequency and compliance with therapy may have also affected the overall attack frequency. We elected not to analyze this as a covariate due to the small sample size and the difficulty of controlling for possible effects of the newly licensed medication availability on the disease burden regardless of medication use frequency.

Second, a substantial risk of recall bias cannot be eliminated, since the questionnaires involved patient self‐reported data which was not confirmed by a third party. Indeed, five patients were unsure of their HAE type, and were, therefore, classified as HAE with unknown type in this study. Finally, this study also included patients with nC1‐INH‐HAE. However, the risk of HAE misdiagnosis in patients with mast cell–mediated angioedema should be low, since patients with mast cell–mediated angioedema do not usually develop gastrointestinal symptoms, which are frequent in HAE, and tend to show a later age at onset, mostly in the 30s. The present study showed no notable difference in gastrointestinal symptoms between patients with HAE Type I/II and those with nC1‐INH‐HAE, or those unsure of their HAE type (Table S3). The mean ± SD age at HAE onset in the present study was 22.2 ± 12.5 years, which is substantially higher compared with age at HAE onset reported in a study from Europe (11.2 ± 7.7 years),^51^ and a study from Europe, Australia, and Canada (11.5 ± 8.9 years).^37^ However, there was no difference in the age at onset between patients with HAE Type I/II and those with nC1‐INH‐HAE or unknown HAE type in this study (Table S3). Moreover, the mean age at HAE onset in Japanese patients reported in previous studies (18.0 [SD, 11.9, *n* = 55] and 24.2 [range 4–68, *n* = 171] years)^40^, ^52^ was also higher versus age at HAE onset reported for patients outside of Japan.

Despite these limitations, most of the adjusted R^2^ scores in the multiple linear regression analysis, except for the AE‐QoL Nutrition domain, were in the range of 0.338–0.520, indicating a good fit of the model.^53^ As the patients may not report all their symptoms to physicians unless specifically asked, or may not even be aware of their symptoms themselves, discovering key clinical manifestations in terms of HRQoL of patients with HAE should contribute to a more precise treatment strategy for individual patients.

## 5 CONCLUSIONS

In the absence of modern LTP options, the frequency of HAE attacks tended to adversely affect many aspects of HRQoL, especially in patients with attacks occurring once a month or more frequently. The AE‐QoL Fatigue/Mood and Fears/Shame domain scores tended to be associated with anxiety as evaluated by the HADS; the AE‐QoL total score and the SF‐12 Physical and Mental Health Composite scores tended to be associated with depression as evaluated by the HADS. Further studies of HRQoL in patients with HAE after modern LTP treatment options become widely available in Japan should be conducted to enable comparison with the results of this study.

## FUNDING INFORMATION

This study was sponsored by Takeda Pharmaceutical Company Limited. Takeda Pharmaceutical Company Limited was involved in designing the study, data collection, data analysis, data interpretation, and provided funding for the editorial assistance.

## CONFLICT OF INTEREST STATEMENT

Michihiro Hide has received advisory fees and honoraria from Takeda, CSL Behring, Torii, KalVista, and Pharvaris. Beverley Anne Yamamoto has received advisory and/or presentation fees from Takeda, CSL Behring, Torii, BioCryst, and KalVista. Miwa Kishimoto, Ippei Kotera, Akinori Oh, and Yoichi Inoue are full‐time employees of Takeda. Shinichi Noto received advisory fees from Takeda. Michihiro Hide, Beverly Anne Yamamoto, and Shinichi Noto did not receive any payment regarding the development of this manuscript from Takeda.

## ETHICS STATEMENT

Ethics approval for this study was obtained from the Ethics Committee of the Non‐Profit Organization, MINS Research Ethics Committee (approval number: MINS‐REC‐200222).

## INFORMED CONSENT STATEMENT

Patients provided agreement to the informed consent statement before the screening. Individuals under the age of 20 were allowed to participate in the survey if both the patient and their caregiver agreed to the informed consent statement.

## STUDY REGISTRATION

The study was registered in the UMIN Clinical Trials Registry (UMIN000042425) before enrollment of the first participant.

## Supporting information


Data S1.

## Data Availability

Takeda does not plan to share the data supporting the results reported in this article as the consent to share the data publicly was not obtained from the study participants.
