# Supplementary material for: Analysis of disease burden in patients with hereditary angioedema from Japan by patient‐reported outcomes
Source: J Dermatol. 2024 Sep 11;52(2):256–69. doi: 10.1111/1346-8138.17421 (PMC11807363; doi:10.1111/1346-8138.17421)
Supplement: Supplementary file 1 — Data S1. [file JDE-52-256-s001.docx]

**Supplementary Material**

**Supplementary figures**

**Figure S1**. 12-Item Short Form Health Survey (SF-12, version 2.0) scores in patients with HAE Type I/II only (excluding the patients with nC1-INH-HAE and patients who were unsure of their HAE type) by frequency of HAE attacks classified into annual, monthly, and weekly. Interquartile range was calculated using the inclusive median. Means are depicted by “x”. HAE, hereditary angioedema; nC1-INH-HAE, hereditary angioedema with normal C1 inhibitor.


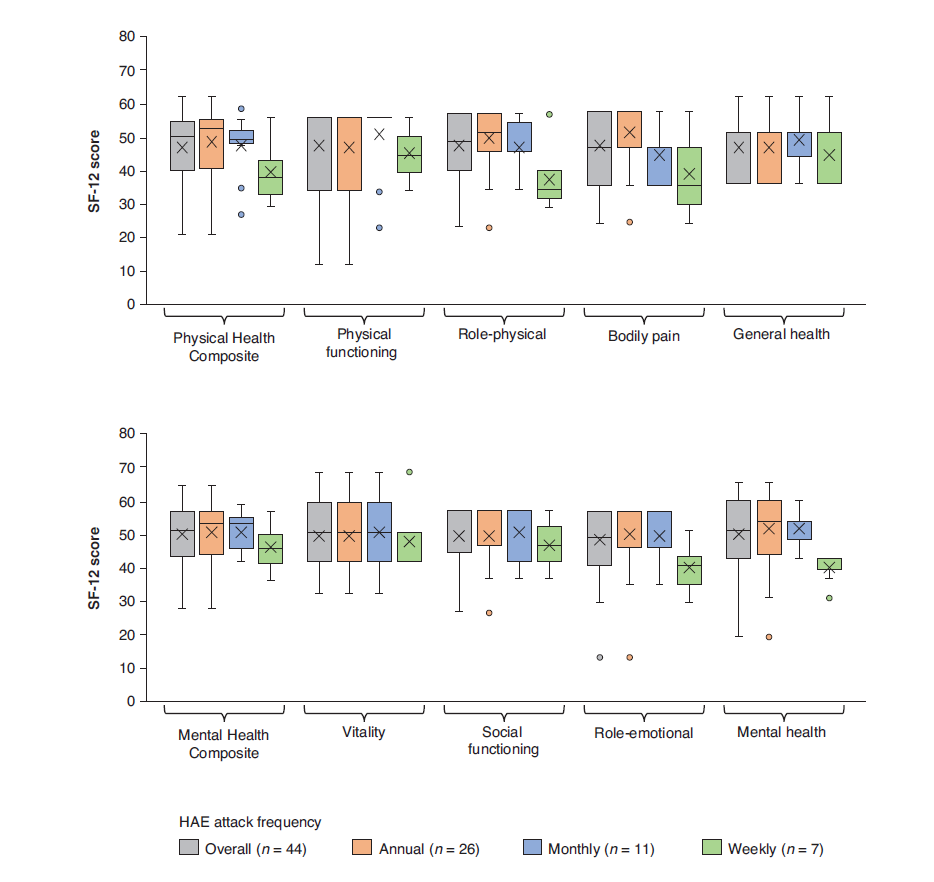


**Figure S2**. Angioedema Quality of Life (AE-QoL) scores in patients with HAE Type I/II only (excluding the patients with nC1-INH-HAE and patients who were unsure of their HAE type) by frequency of HAE attacks classified into annual, monthly, and weekly. Interquartile range was calculated using the inclusive median. Means are depicted by “x”. HAE, hereditary angioedema; nC1-INH-HAE, hereditary angioedema with normal C1 inhibitor.


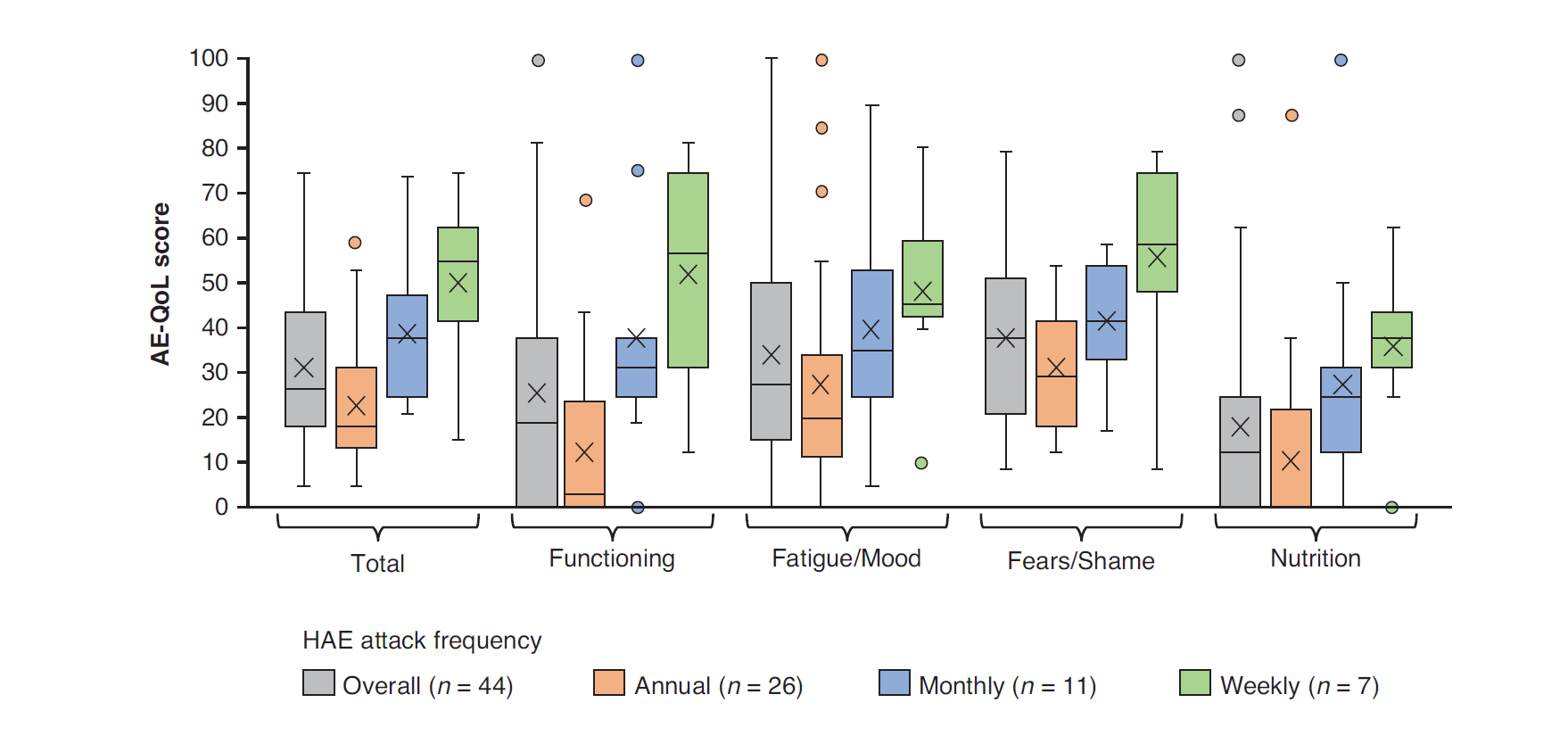


**Figure S3.** Hospital Anxiety and Depression Scale (HADS) scores in patients with HAE Type I/II only (excluding the patients with nC1-INH-HAE and patients who patients who were unsure of their HAE type) by frequency of HAE attacks classified into annual, monthly, and weekly. Interquartile range was calculated using the inclusive median. Means are depicted by “x”. HAE, hereditary angioedema; nC1-INH-HAE, hereditary angioedema with normal C1 inhibitor.


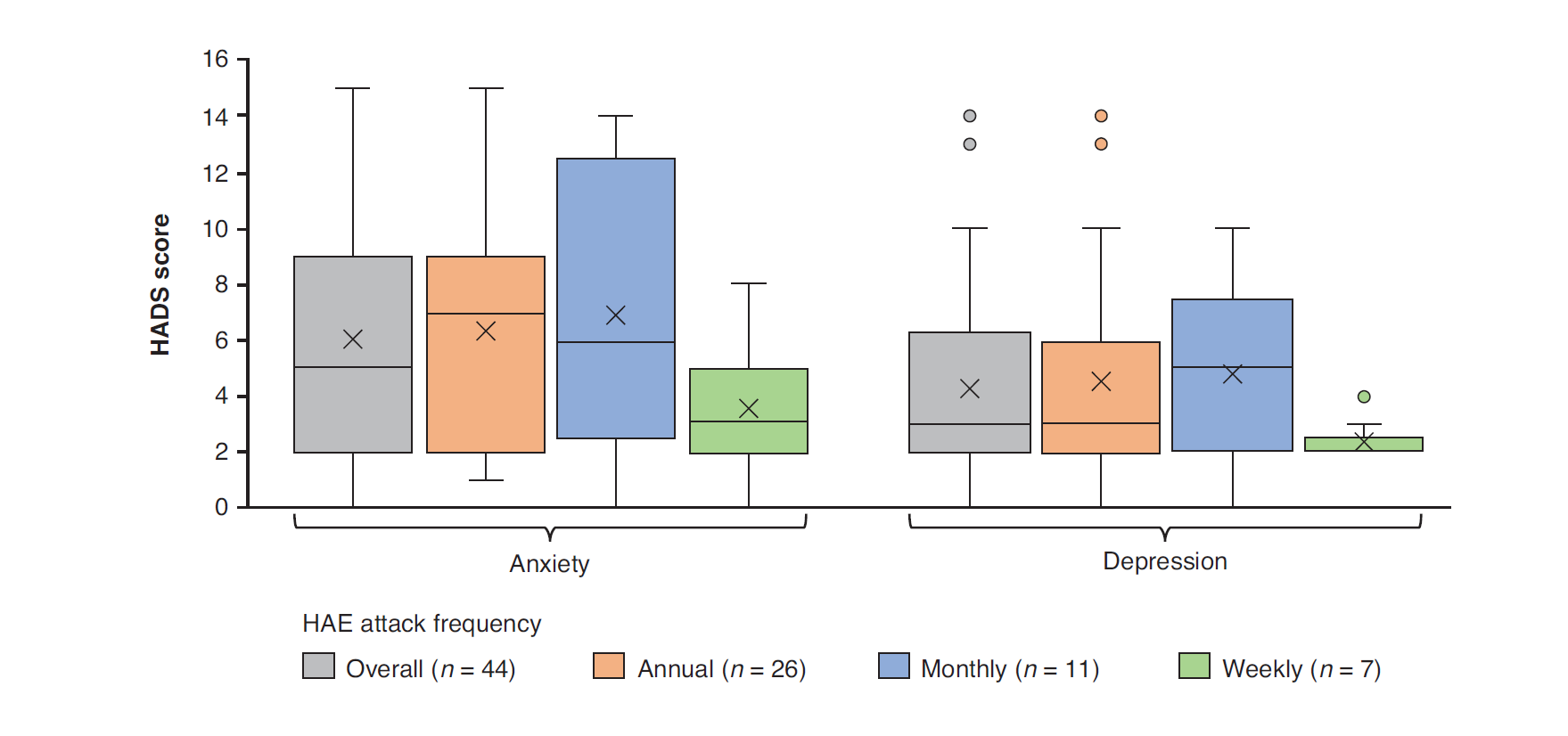


**Figure S4.** Work Productivity and Activity Impairment: Specific Health Problem V2.0 (WPAI:SHP) scores in patients with HAE Type I/II only (excluding the patients with nC1-INH-HAE and patients who patients who were unsure of their HAE type) by frequency of HAE attacks classified into annual, monthly, and weekly. Percentage impairment in absenteeism, presenteeism, and work productivity loss was calculated for 20 patients with HAE Type I/II with any employment. Percentage impairment in activity impairment was calculated for all patients with HAE Type I/II in the survey and reported separately in employed and unemployed patients. Interquartile range was calculated using the inclusive median. Means are depicted by “x”. HAE, hereditary angioedema; nC1-INH-HAE, hereditary angioedema with normal C1 inhibitor.


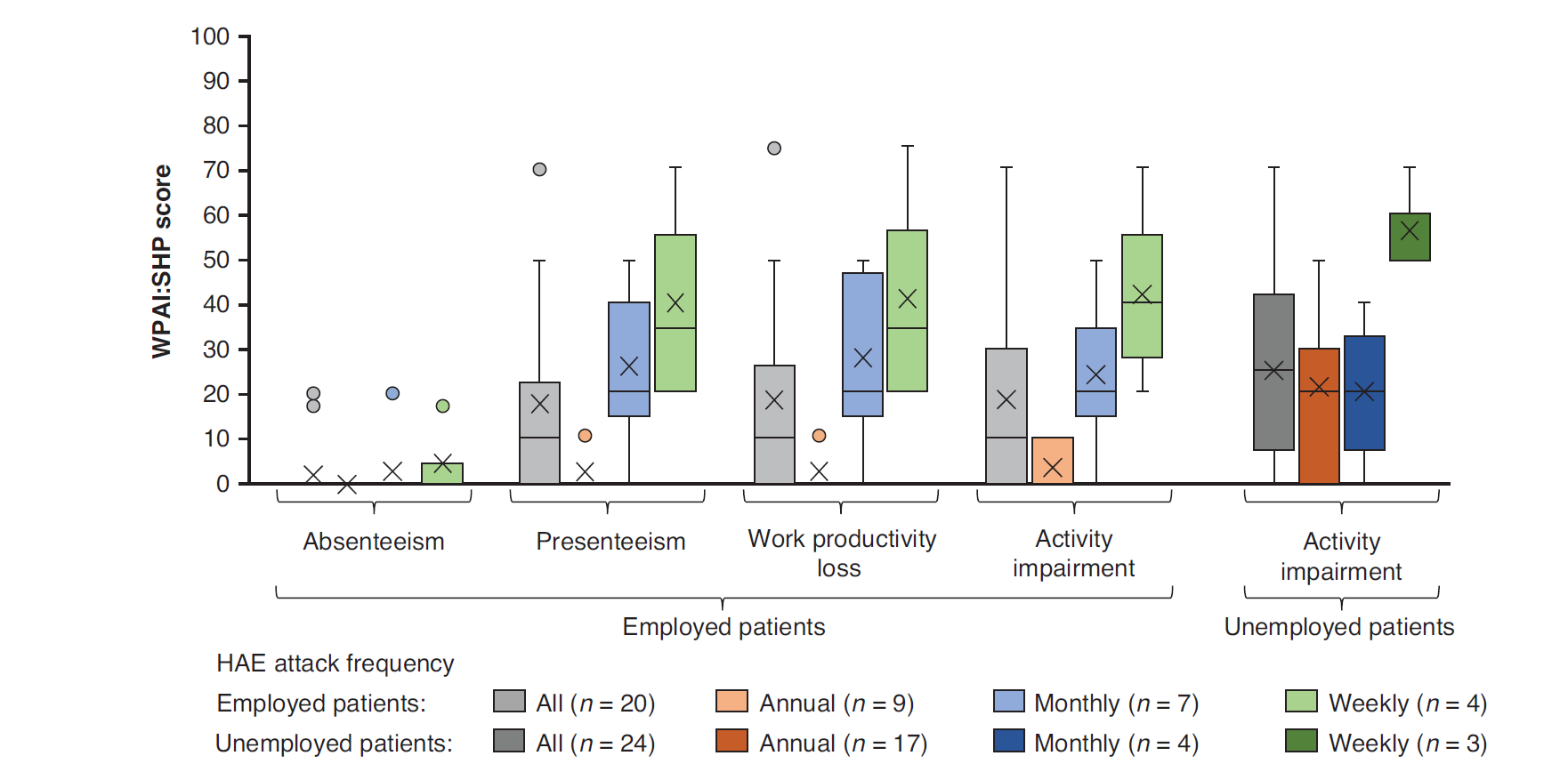


**Figure S5.** SF-12 Physical Health Composite scores in patients with HAE grouped by the presence or absence of gastrointestinal symptoms. Interquartile range was calculated using inclusive median. Means are depicted by “x”. HAE, hereditary angioedema; SF-12, 12-Item Short Form Health Survey (version 2.0).


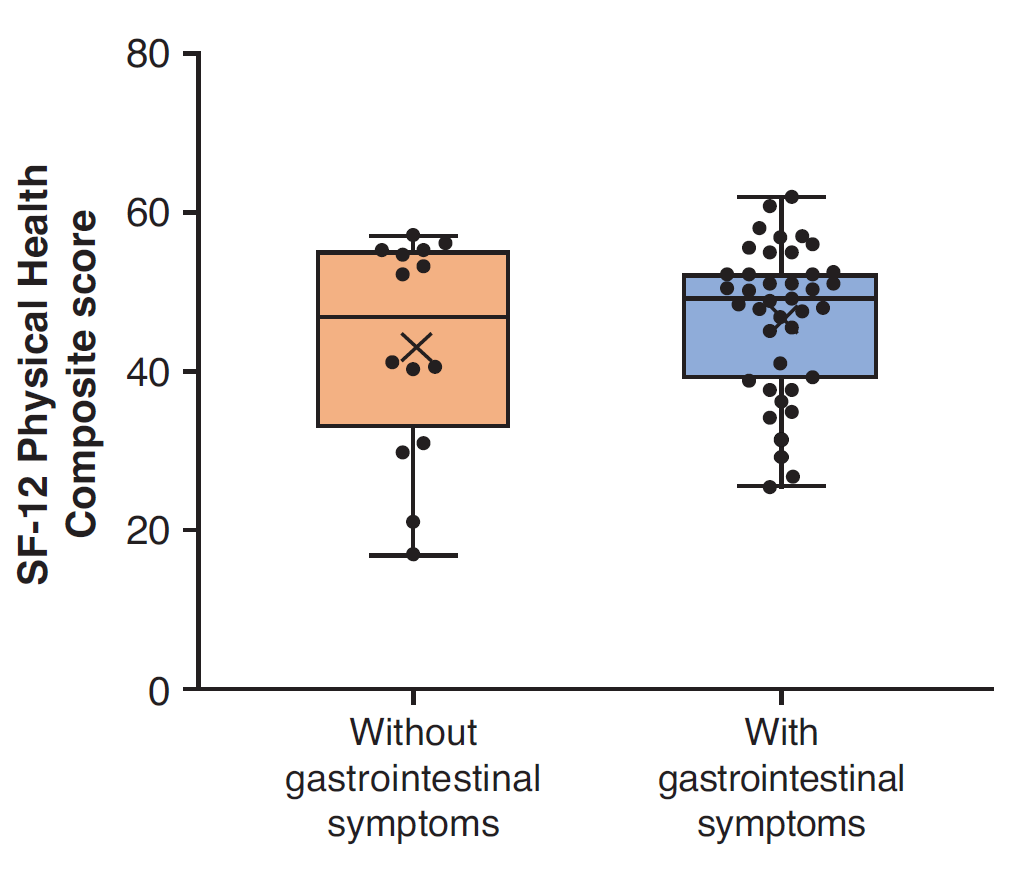


**Supplementary tables**

**Table S1.** PROMs used in this study

| **PROM** | **Description** |
| --- | --- |
| 12-Item Short-Form Health Survey, version 2.0 (SF-12) | - A generic PROM assessing general health^1^ - Has 12 questions in eight domains (physical functioning, role-physical, bodily pain, general health, vitality, social functioning, role-emotional, and mental health) as well as two summary scales (Physical Health Composite and Mental Health Composite)^1^ - Has a recall period “during the last four weeks”^1^ - Domain and summary scores have a range of 0–100; higher scores reflect better health, and a score of 50 reflects the standard score of the general population^1^ - SF-12 Mental Health Composite score of ≤42 has been suggested to be indicative of clinical depression^2^ |
| Angioedema Quality of Life (AE-QoL) | - Disease-specific PROM assessing HRQoL impairment in patients with recurrent angioedema^3^ - Validated in adult patients with HAE^4^ - Validated in Japanese^5^ - Has 17 questions in four domains (Functioning, Fatigue/Mood, Fears/Shame, and Nutrition), and a total score^3^ - Has a recall period “over the past four weeks”^3^ - Domain and total scores have a range of 0–100; higher scores reflect greater HRQoL impairment^3^ - A cutoff of ≥39 points in the total score reflects moderate to large HRQoL impairment^6^ |
| Hospital Anxiety and Depression Scale (HADS) | - A generic PROM assessing anxiety and depression^7^ - Has 14 questions (seven for anxiety, seven for depression)^7^ - Has a recall period “in the past week”^7^ - Subscale scores have a range of 0–21; higher scores reflect greater impairment^7^ - Subscale scores of 0–7 suggest normal levels of anxiety/depression, scores of 8–10 suggest mild anxiety/depression, scores of 11–14 suggest moderate anxiety/depression, and scores of 15–21 suggest severe anxiety/depression^8^ - HADS total score can be calculated by summing all HADS items and has a range of 0–42, where higher scores reflect greater psychological distress^9^ |
| Work Productivity and Activity Impairment: Specific Health Problem (WPAI:SHP) | - A PROM assessing the effect of specific health problem on the patients’ ability to work/perform regular activities^10,11^ - Has six questions used to calculate percentage impairment in four domains (presenteeism: work time missed; absenteeism: reduced work productivity while working; overall work productivity loss: combined absenteeism and presenteeism; and activity impairment: loss of productivity in general daily nonwork activities)^10,11^ - Has a recall period “during the past seven days”^10,11^ - Higher domain scores reflect greater productivity impairment^10,11^ |
| Angioedema Activity Score (AAS) | - Disease-specific PROM assessing angioedema activity in patients with recurrent angioedema^12^ - Validated in Japanese^5^ - Has five questions^12^ - Has a recall period “during the past 24 hours” - Scores have a range of 0–15; higher scores reflect higher angioedema activity^12^ |

PROMs, patient-reported outcome measures.

**Table S2.** Detailed demographic and clinical characteristics

|  |  | **Frequency of HAE attacks** | | |
| --- | --- | --- | --- | --- |
|  | **All (N=54)** | **Annual^†^ (n=30)** | **Monthly^‡^ (n=15)** | **Weekly^§^ (n=9)** |
| Age (years), n (%) |  |  |  |  |
| 19 | 0 (0.0) | 0 (0.0) | 0 (0.0) | 0 (0.0) |
| 20–29 | 1 (1.9) | 1 (3.3) | 0 (0.0) | 0 (0.0) |
| 30–39 | 13 (24.1) | 6 (20.0) | 4 (26.7) | 3 (33.3) |
| 40–49 | 18 (33.3) | 8 (26.7) | 7 (46.7) | 3 (33.3) |
| 50–59 | 11 (20.4) | 8 (26.7) | 2 (13.3) | 1 (11.1) |
| 60+ | 11 (20.4) | 7 (23.3) | 2 (13.3) | 2 (22.2) |
| Age at onset (years), n (%) |  |  |  |  |
| 0–9 | 5 (9.3) | 1 (3.3) | 1 (6.7) | 3 (33.3) |
| 10–19 | 17 (31.5) | 9 (30.0) | 4 (26.7) | 4 (44.4) |
| 20–29 | 20 (37.0) | 11 (36.7) | 8 (53.3) | 1 (11.1) |
| 30–39 | 7 (13.0) | 5 (16.7) | 1 (6.7) | 1 (11.1) |
| 40–49 | 3 (5.6) | 2 (6.7) | 1 (6.7) | 0 (0.0) |
| 50–59 | 0 (0.0) | 0 (0.0) | 0 (0.0) | 0 (0.0) |
| 60+ | 2 (3.7) | 2 (6.7) | 0 (0.0) | 0 (0.0) |
| Age at diagnosis (years), n (%) |  |  |  |  |
| 0–9 | 1 (1.9) | 0 (0.0) | 0 (0.0) | 1 (11.1) |
| 10–19 | 3 (5.6) | 2 (6.7) | 0 (0.0) | 1 (11.1) |
| 20–29 | 9 (16.7) | 3 (10.0) | 4 (26.7) | 2 (22.2) |
| 30–39 | 19 (35.2) | 10 (33.3) | 6 (40.0) | 3 (33.3) |
| 40–49 | 14 (25.9) | 10 (33.3) | 4 (26.7) | 0 (0.0) |
| 50–59 | 5 (9.3) | 3 (10.0) | 0 (0.0) | 2 (22.2) |
| 60+ | 3 (5.6) | 2 (6.7) | 1 (6.7) | 0 (0.0) |
| Site or area of HAE attacks^¶^, n (%) |  |  |  |  |
| Face | 25 (46.3) | 10 (33.3) | 8 (53.3) | 7 (77.8) |
| Lips | 32 (59.3) | 15 (50.0) | 9 (60.0) | 8 (88.9) |
| Around the eyes | 24 (44.4) | 14 (46.7) | 5 (33.3) | 5 (55.6) |
| Nose | 8 (14.8) | 3 (10.0) | 2 (13.3) | 3 (33.3) |
| Tongue | 10 (18.5) | 1 (3.3) | 4 (26.7) | 5 (55.6) |
| Hand | 38 (70.4) | 20 (66.7) | 10 (66.7) | 8 (88.9) |
| Foot | 36 (66.7) | 20 (66.7) | 8 (53.3) | 8 (88.9) |
| Arm | 30 (55.6) | 16 (53.3) | 8 (53.3) | 6 (66.7) |
| Trunk | 14 (25.9) | 2 (6.7) | 6 (40.0) | 6 (66.7) |
| Genitals | 19 (35.2) | 6 (20.0) | 5 (33.3) | 8 (88.9) |
| None of the above | 9 (16.7) | 7 (23.3) | 1 (6.7) | 1 (11.1) |
| Family history, n (%) |  |  |  |  |
| Yes | 35 (64.8) | 20 (66.7) | 9 (60.0) | 6 (66.7) |
| No | 19 (35.2) | 10 (33.3) | 6 (40.0) | 3 (33.3) |
| Treatment medications^¶^, n (%) |  |  |  |  |
| Tranexamic acid | 28 (51.9) | 14 (46.7) | 9 (60.0) | 5 (55.6) |
| Androgen | 2 (3.7) | 0 (0.0) | 0 (0.0) | 2 (22.2) |
| C1-INH replacement therapy^††^ | 36 (66.7) | 17 (56.7) | 11 (73.3) | 8 (88.9) |
| Blood preparation other than C1-INH | 4 (7.4) | 2 (6.7) | 1 (6.7) | 1 (11.1) |
| Icatibant | 21 (38.9) | 7 (23.3) | 9 (60.0) | 5 (55.6) |
| Investigational drug in clinical trial | 0 (0.0) | 0 (0.0) | 0 (0.0) | 0 (0.0) |
| Other drugs | 5 (9.3) | 2 (6.7) | 3 (20.0) | 0 (0.0) |
| No treatment | 3 (5.6) | 3 (10.0) | 0 (0.0) | 0 (0.0) |
| Prophylactic agents^¶^, n (%) |  |  |  |  |
| Tranexamic acid | 33 (61.1) | 17 (56.7) | 10 (66.7) | 6 (66.7) |
| Androgen | 4 (7.4) | 1 (3.3) | 1 (6.7) | 2 (22.2) |
| C1-INH replacement therapy | 16 (29.6) | 5 (16.7) | 5 (33.3) | 6 (66.7) |
| Investigational drug in clinical trial | 0 (0.0) | 0 (0.0) | 0 (0.0) | 0 (0.0) |
| Other drugs | 8 (14.8) | 1 (3.3) | 6 (40.0) | 1 (11.1) |
| No treatment | 10 (18.5) | 8 (26.7) | 1 (6.7) | 1 (11.1) |
| Comorbidities (skin)^¶^, n (%) |  |  |  |  |
| Urticaria | 5 (9.3) | 1 (3.3) | 2 (13.3) | 2 (22.2) |
| Other skin disorders | 7 (13.0) | 3 (10.0) | 2 (13.3) | 2 (22.2) |
| None | 44 (81.5) | 26 (86.7) | 12 (80.0) | 6 (66.7) |
| Comorbidities (autoimmune disease)^¶^, n (%) |  |  |  |  |
| Systemic lupus erythematosus | 1 (1.9) | 1 (3.3) | 0 (0.0) | 0 (0.0) |
| Thyroiditis (Hashimoto’s disease) | 0 (0.0) | 0 (0.0) | 0 (0.0) | 0 (0.0) |
| Glomerulonephritis | 0 (0.0) | 0 (0.0) | 0 (0.0) | 0 (0.0) |
| Inflammatory bowel disease | 1 (1.9) | 0 (0.0) | 0 (0.0) | 1 (11.1) |
| Sjögren’s syndrome | 0 (0.0) | 0 (0.0) | 0 (0.0) | 0 (0.0) |
| Other autoimmune disorders | 3 (5.6) | 1 (3.3) | 1 (6.7) | 1 (11.1) |
| None | 49 (90.7) | 28 (93.3) | 14 (93.3) | 7 (77.8) |
| History of malignancy, n (%) |  |  |  |  |
| Yes | 1 (1.9) | 1 (3.3) | 0 (0.0) | 0 (0.0) |
| No | 53 (98.1) | 29 (96.7) | 15 (100.0) | 9 (100.0) |
| Psychiatric disorders, n (%) |  |  |  |  |
| Yes | 4 (7.4) | 1 (3.3) | 1 (6.7) | 2 (22.2) |
| No | 50 (92.6) | 29 (96.7) | 14 (93.3) | 7 (77.8) |
| Comorbidities^¶^, n (%) |  |  |  |  |
| Hypertension | 5 (9.3) | 3 (10.0) | 1 (6.7) | 1 (11.1) |
| Hyperlipidemia | 6 (11.1) | 4 (13.3) | 0 (0.0) | 2 (22.2) |
| Diabetes mellitus | 5 (9.3) | 3 (10.0) | 1 (6.7) | 1 (11.1) |
| Heart disease | 1 (1.9) | 1 (3.3) | 0 (0.0) | 0 (0.0) |
| Stroke | 0 (0.0) | 0 (0.0) | 0 (0.0) | 0 (0.0) |
| Anemia | 0 (0.0) | 0 (0.0) | 0 (0.0) | 0 (0.0) |
| Arthritis | 6 (11.1) | 4 (13.3) | 1 (6.7) | 1 (11.1) |
| Emphysema and COPD | 0 (0.0) | 0 (0.0) | 0 (0.0) | 0 (0.0) |
| Asthma | 6 (11.1) | 4 (13.3) | 2 (13.3) | 0 (0.0) |
| Sleep apnea syndrome | 1 (1.9) | 1 (3.3) | 0 (0.0) | 0 (0.0) |
| None | 37 (68.5) | 20 (66.7) | 11 (73.3) | 6 (66.7) |

^†^Annual: patients with several HAE attacks annually.

^‡^Monthly: patients with several HAE attacks monthly.

^§^Weekly: patients with several HAE attacks weekly.

^¶^Multiple responses were allowed.

^††^C1-INH intravenously for on-demand treatment or short-term prophylaxis.
C1-INH, C1 esterase inhibitor; COPD, chronic obstructive pulmonary disease; HAE, hereditary angioedema; SD, standard deviation

**Table S3.** Sex, age, gastrointestinal symptoms, and AE-QoL by HAE type

|  |  | | |  |  | |  |  | |  |  |  | | **AE-QoL** | |  | |  | |
| --- | --- | --- | --- | --- | --- | --- | --- | --- | --- | --- | --- | --- | --- | --- | --- | --- | --- | --- | --- |
|  | **Sex** | | |  | **Age** | |  | **Gastrointestinal symptoms** | |  | **Functioning** | | **Fatigue /Mood** | | **Fears /Shame** | | **Nutrition** | | **Total** |
| **Type of HAE** | **Male** | **Female** | **Total** |  | **Age at study, mean ± SD, years** | **Age at onset, mean± SD, years** |  | **Total (%)** | **Female (%)** |  | **Mean ± SD** | | **Mean ± SD** | | **Mean ± SD** | | **Mean ± SD** | | **Mean ± SD** |
| I/II | 4 | 40 | 44 |  | 48.8 ± 11.7 | 22.3 ± 12.8 |  | 31 (70.5) | 38  (95.0) |  | 25.1 ± 26.3 | | 27.2 ± 19.9 | | 20.3 ± 11.4 | | 9.2 ± 11.9 | | 22.1 ± 14.3 |
| Normal  C1-INH | 0 | 5 | 5 |  | 42.0 ± 8.7 | 24.8 ± 15.9 |  | 4 (80.0) | 4  (80.0) |  | 60.0 ± 22.6 | | 22.7 ± 30.8 | | 30.8 ± 12.8 | | 25.0 ± 13.7 | | 40.6 ± 9.2 |
| Unknown | 3 | 2 | 5 |  | 47.2 ± 18.9 | 18.2 ± 5.9 |  | 5 (100.0) | 2  (100.0) |  | 32.5 ± 18.3 | | 32.0 ± 13.6 | | 24.2 ± 3.1 | | 7.5 ± 7.3 | | 26.5 ± 6.6 |

AE-QoL, Angioedema Quality of Life; C1-INH, C1 esterase inhibitor; HAE, hereditary angioedema; SD, standard deviation.

**Supplementary references**

1. Ware J, Jr., Kosinski M, Keller SD. A 12-item short-form health survey: construction of scales and preliminary tests of reliability and validity. Med Care. 1996;34(3):220-33.

2. Ware J. E. J, Kosinski M, Keller SD. SF-12: How to score the SF-12 Physical and Mental Summary Scales*,* 2nd edn. Boston: The Health Institute, New England Medical Center, 1995.

3. Weller K, Groffik A, Magerl M, Tohme N, Martus P, Krause K, et al. Development and construct validation of the angioedema quality of life questionnaire. Allergy. 2012;67(10):1289–98.

4. Vanya M, Watt M, Shahraz S, Kosmas CE, Rhoten S, Costa-Cabral S, et al. Content validation and psychometric evaluation of the Angioedema Quality of Life Questionnaire for hereditary angioedema. J Patient Rep Outcomes. 2023;7(1):33.

5. Morioke S, Takahagi S, Kawano R, Fukunaga A, Harada S, Ohsawa I, et al. A validation study of the Japanese version of the Angioedema Activity Score (AAS) and the Angioedema Quality of Life Questionnaire (AE-QoL). Allergol Int. 2021;70(4):471–9.

6. Kulthanan K, Chularojanamontri L, Rujitharanawong C, Weerasubpong P, Maurer M, Weller K. Angioedema quality of life questionnaire (AE-QoL) - interpretability and sensitivity to change. Health Qual Life Outcomes. 2019;17(1):160.

7. Zigmond AS, Snaith RP. The hospital anxiety and depression scale. Acta Psychiatr Scand. 1983;67(6):361–70.

8. Stern AF. The hospital anxiety and depression scale. Occup Med (Lond). 2014;64(5):393–4.

9. Hyland KA, Hoogland AI, Gonzalez BD, Nelson AM, Lechner S, Tyson DM, et al. Evaluation of the psychometric and structural properties of the Spanish version of the Hospital Anxiety and Depression Scale in Latina cancer patients. J Pain Symptom Manage. 2019;58(2):289–96.

10. Reilly Associates. Work Productivity and Activity Impairment Questionnaire: Specific Health Problem V2.0 (WPAI:SHP). 2010. 2023. Available from: <http://www.reillyassociates.net/WPAI_SHP.html>

11. Reilly MC, Zbrozek AS, Dukes EM. The validity and reproducibility of a work productivity and activity impairment instrument. Pharmacoeconomics. 1993;4(5):353–65.

12. Weller K, Groffik A, Magerl M, Tohme N, Martus P, Krause K, et al. Development, validation, and initial results of the Angioedema Activity Score. Allergy. 2013;68(9):1185–92.
